# Audit of a computerized version of the Manchester triage system and a SIRS-based system for the detection of sepsis at triage in the emergency department

**DOI:** 10.1186/s12245-022-00472-y

**Published:** 2022-12-13

**Authors:** Ken Dewitte, Elyne Scheurwegs, Sabrina Van Ierssel, Hilde Jansens, Karolien Dams, Ella Roelant

**Affiliations:** 1 Emergency Department, Antwerp University Hospital, Edegem, Belgium; 2 ADREM (Advanced Database Research and Modelling), Biomedical Informatics Research Center Antwerp (Biomina), University of Antwerp, Antwerpen, Belgium; 3 Department of General Internal Medicine, infectious diseases and tropical medicine, Antwerp University Hospital, Edegem, Belgium; 4 Department of Infection Control and Microbiology, Antwerp University Hospital, Edegem, Belgium; 5 Department of Intensive Care Medicine, Antwerp University Hospital, Edegem, Belgium; 6 Clinical Trial Center (CTC), Clinical Research Center Antwerp, Antwerp University Hospital, Edegem, Belgium

**Keywords:** Manchester triage system, Sepsis, Emergency department

## Abstract

**Background and importance:**

Different triage systems can be used to screen for sepsis and are often incorporated into local electronic health records. Often the design and interface of these digitalizations are not audited, possibly leading to deleterious effects on screening test performance.

**Objective:**

To audit a digital version of the MTS for detection of sepsis during triage in the ED.

**Design:**

A single-center retrospective study

**Settings and participants:**

Patients (*n*=29766) presenting to an ED of a tertiary-care center who received formal triage were included.

**Outcome measures and analysis:**

Calculated performance measures included sensitivity, specificity, likelihood ratios, and AUC for the detection of sepsis. Errors in the application of the specific sepsis discriminator of the MTS were recorded.

**Main results:**

A total of 189 (0.7%) subjects met the Sepsis-3 criteria, with 47 cases meeting the criteria for septic shock. The MTS had a low sensitivity of 47.6% (95% CI 40.3 to 55.0) for allocating sepsis patients to the correct triage category. However, specificity was high at 99.4% (95% CI 99.3 to 99.5).

## Introduction

Numerous studies explored how sepsis could be detected as early as possible after the presentation to the emergency department to prioritize treatment of these patients using computerized decision support systems [1, 2]. NEWS, qSOFA, SIRS, Manchester Triage System, ATS (Australian Triage Scale), CTAS (Canadian Acuity Triage Scale), and ESI (Emergency Severity Index) are among the most used scoring systems during triage, and there is a wide variation in diagnostic accuracy of these tools [3–11]. The Manchester Triage System (MTS) is a 5-level triage system commonly used in Europe. This algorithm uses flowcharts describing the signs and symptoms of the patients, such as “general unwell being” and “abdominal pain.” MTS priorities range from level 1 (emergent patients that should have immediate medical care) to level 5 (non-urgent patients that could wait a maximum of 4 hours to be seen) [12].

**Fig. 1:**
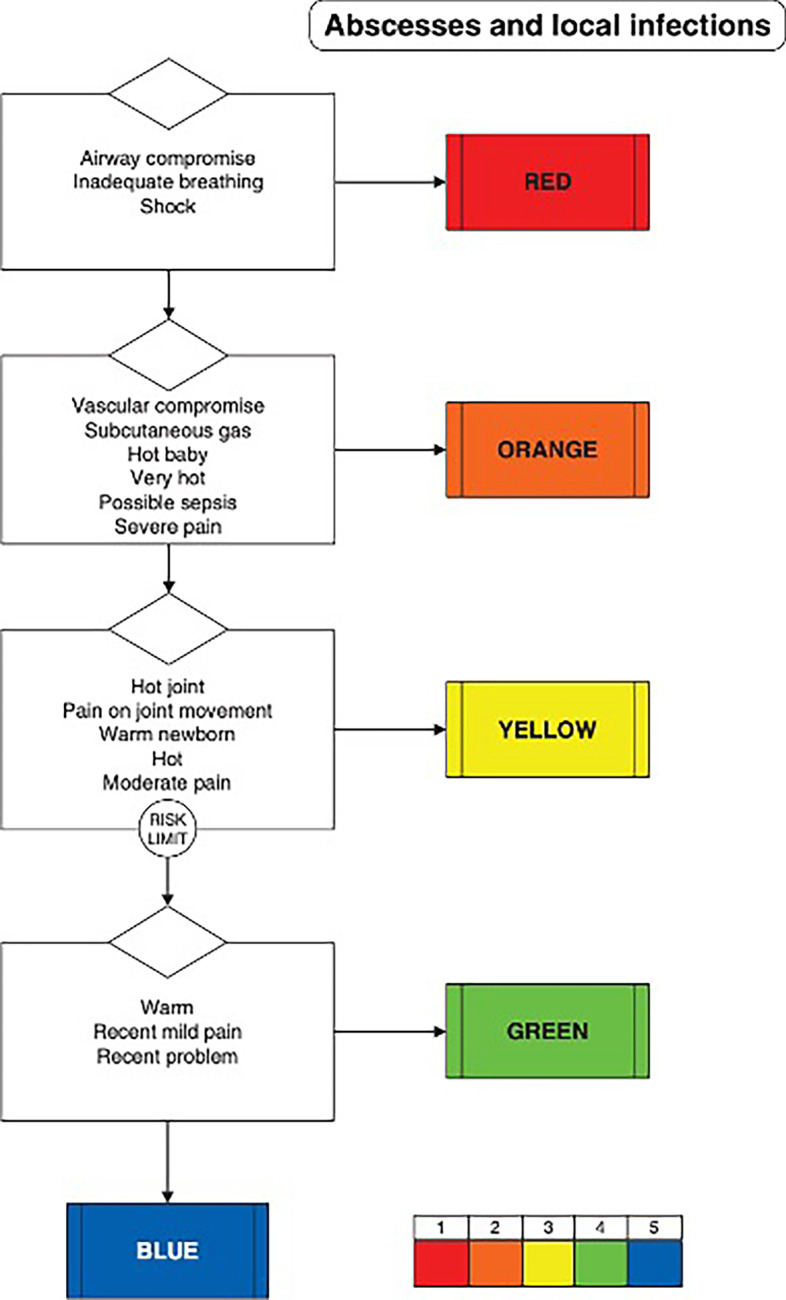
Example of MTS flow chart

A specific discriminator for possible sepsis was recently added to the Manchester triage system (https://www.triagenet.net/en/files/MTSETUpdates.pdf). In the current version of the MTS, possible sepsis is defined as a patient that meets one or more qSOFA criteria. See Fig. 1 for an example.

Seymour et al. introduced the quick Sequential Organ Failure Assessment (qSOFA) score to rapidly identify patients with suspected infection at risk for sepsis outside the ICU [13]. It is a simple score consisting of three items: respiratory rate (RR) ≥ 22 breaths per minute, altered mentation (Glasgow Coma Scale [GCS] < 15), and systolic blood pressure (SBP) < 100 mmHg. A qSOFA score ≥ 2 was found to be significantly predictive of increased all-cause mortality in patients outside of the ICU. Therefore, it was introduced as a score for detecting patients at risk for sepsis. However, recently, the surviving sepsis campaign advised against using the qSOFA as a single screening tool compared with SIRS or NEWS to identify sepsis [14].

In most recent studies and in the clinical setting, the MTS system is being used in a locally developed computerized versions and often additional features or important modifications are made [15]. Above this, the Royal College of Emergency Medicine raised a concern following several reported incidents that organisations may not be using the latest version of the system [16].

We hypothesized important disparities and errors can be made due to the inappropriate design of these computerized systems.

In this paper, we audit the digital version of a locally developed software program of the MTS and a SIRS-based sepsis screening tool in a busy emergency department on a real-world dataset.

## Methods

### Study design and definitions

Retrospective analysis of all adult patients (>18 years of age) presenting to the emergency department from January 2020 to June 2021 who received a formal triage.

The ED of the Antwerp University Hospital is a level 1 trauma center receiving about 30,000 patients annually.

Triage in the ED at our hospital is a standardized process in which triage nurses trained in using the Manchester Triage System evaluate each patient that enters the ED. Data such as vital signs are manually entered, and a SIRS-based sepsis screening tool has to be filled out for every patient into the electronic health record (EHR) (C2M, Cegeka Belgium).

Sepsis and septic shock were defined as per Sepsis-3 definitions: a Sequential Organ Failure Assessment (SOFA) score of ≥2 and suspected infection [13]. Suspicion of infection was determined by orders for administering antibiotics or blood cultures.

Patients with sepsis categorized as red, orange or “possible sepsis” were considered correctly triaged. The proportion of correctly triaged and the different diagnostic performance measures sensitivity, specificity, positive and negative likelihood ratios, and accuracy were calculated.

### Sepsis screening tools used in our ED during triage

At our center, each patient admitted to the ED is being screened for sepsis using the MTS and a SIRS-based screening tool.

A trained nurse follows the process described by MTS to define a priority code. The priority codes are divided into five levels of urgency: blue (non-urgent, 240 min), green (average, 120 min), yellow (urgent, 60 min), orange (very urgent, 10 min), and red (immediate, 0 min).

**Fig. 2:**
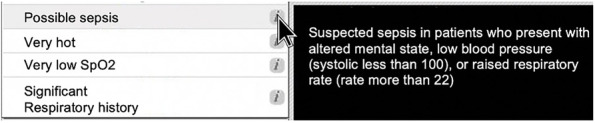
Screenshot interface digital MTS flow chart

During the triage evaluation, the nurse chooses the appropriate MTS diagram and discriminator. In our digital version, the explanation for each specific discriminator, including for “possible sepsis” is visualized when hovering over a specific symbol. See example Fig. 2.

It is important to note that vital signs data are not cross-checked with the digital Manchester Triage tool being used during this trial due to software-related limitations.

**Fig. 3:**
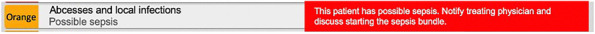
Screenshot digital sepsis alert

A digital sepsis alert is generated if the triage nurse chooses the MTS category “possible sepsis” in our system. See Fig. 3.

**Fig. 4:**
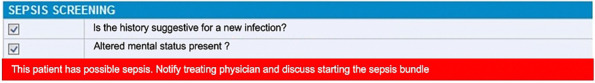
Screenshot interface digital SIRS-based screening tool and alert

Our EHR has a separate SIRS-based sepsis screening tool using the sepsis-2 criteria. A digital sepsis alert is fired only if the triage nurse determines there is a suspicion of a new infection in combination with one of the two following: an altered mental state (separate checkbox) or two or more selected SIRS criteria (temperature less than 36 or more than 38 degrees Celsius, heart rate more than 90 beats per minute, a respiratory rate more than 20 per minute). Contrary to the MTS, triggers were programmed to fire the alert based on values of temperature, heart rate, or respiratory rate entered in the EHR. See Fig. 4.

### Study population

All adult patients (>18 years of age) presenting to the emergency department that received a formal triage were included (*n*=29766). Patients assigned to the category “blue” were excluded from further analysis (*n*=1537) as no case of suspected or confirmed sepsis did occur in this category (no blood cultures ordered, no “possible sepsis” marked in the digital Manchester Triage system, no admittance to ICU and no case meeting sepsis criteria after admission). Also, patients that received immediate palliative care after diagnosis of possible sepsis in the ED were excluded (*n*=16), leaving 28,213 patients for further analysis.

### Data management and patient chart data review

Data was extracted as-is from the EHR. The patient’s state is represented as the earliest measurement known for each patient during their stay at the ED. This approximates the state of the patient as presented first in the ED.

The total number of missing data for calculating screening scores was relatively low (975 missing SIRS criteria and 402 observations rendering the calculation of qSOFA impossible (33 missing blood pressure recordings, 369 missing mental status scores). In agreement with clinical practice and prior reports, missing data for SOFA score calculation were assumed to be normal [17].

An arbitrary selection of records for in-depth review was made based on the presence of at least one or more of the following criteria: categorization to “possible sepsis” in the MTS, NEWS score >5, documentation of “suspicion of infection” in the triage chart, one or more qSOFA criteria, a positive SIRS-based sepsis screening, categorization to “red” in the MTS, blood cultures taken, admission to ICU within 72 h after admission on ED or death during a hospital stay. An emergency physician (author KD) manually reviewed these selected patient charts (*n*=725) to verify the sepsis diagnosis, the triage system’s correct application, and the accuracy of data input.

For example, if the respiratory rate was marked as normal, but clinical examination mentioned tachypnea or blood gases showed clear hyperventilation, this data was flagged as incorrect. Mental status scoring was derived from the separate question that was asked in the SIRS-based screening software in which a checkbox was used to confirm an altered mental status.

Of these records, two independent emergency physicians not involved in the main study reviewed 30 random cases to calculate inter-rater agreement for triage categorization and sepsis diagnosis, which was moderate to high (weighted kappa 0.41 and 0.86).

### Endpoint

The primary endpoint was the diagnostic accuracy of the MTS and the SIRS-based tool to identify sepsis at triage. In a subsequent analysis, we compared the performance of a combination of the MTS with a SIRS-based sepsis screening tool.

### Statistical analysis

Sample size calculation showed that given a prevalence of 0.5% of sepsis patients and a supposed sensitivity of 80% and specificity of 98% at an estimation error of 8%, the minimum sample size was 19400 and a minimum number of 97 sepsis patients [18].

Baseline characteristics between different triage categories were compared using ANOVA analysis or chi-square test as appropriate. Sensitivity, specificity, positive and negative predictive values, positive and negative likelihood ratios, and AUC values were calculated using a two × two contingency table. Finally, the sensitivity and specificity of each model were compared using McNemar’s test [19].

All estimates are presented with their 95% confidence intervals, and a *p* value of less than 0.05 was considered statistically significant for all analyses. MedCalc Statistical Software version 19.2.6 (MedCalc Software bv, Ostend, Belgium; https://www.medcalc.org; 2020) was used for calculations.

## Results

A total of 189 (0.7%) subjects met the Sepsis-3 criteria, with 47 cases meeting the criteria for septic shock. See Table 1 for baseline characteristics.

**Table 1:** Baseline characteristics of the study population

***Category***	***Green***	***Yellow***	***Orange***	***"Possible sepsis" category***	***Red***	***Total***	***Sign. Level difference between categories***
***Number of patients, n***	*13250*	*11040*	*3363*	*231*	*329*	*28213*	*n/a*
***Mean age (range)***	*45 (30–62)*	*51 (33–67)*	*59 (41–73)*	*60.5 (49–74)*	*64 (50–75)*	*50 (33–66)*	*p<0.001*
***Sepsis diagnosis, n***	*9*	*45*	*25*	*45*	*18*	*142*	*p<0.001*
***Septic shock diagnosis, n***	*1*	*6*	*13*	*14*	*13*	*47*	*p<0.001*
***Mean NEWS score (SD)***	*0.6 (0.9)*	*1.1 (1.3)*	*2.0 (2.3)*	*5.2 (2.8)*	*3.8 (3.9)*	*1.0 (0.0)*	*p<0.001*
***SOFA score sepsis patients (range)***	*5.1 (2–10)*	*4.3 (2–12)*	*6.1 (2–18)*	*5.8 (2–18)*	*9.7 (2–25)*	*6.1 (2–25)*	*p<0.001*
***ICU admission in sepsis, n (% of total sepsis per MTS category)***^a^	*0 (0)*	*7 (15.6)*	*9 (30.6)*	*9 (20.0)*	*16 (88.9)*	*41 (28.9)*	*p=2.17*
***ICU admission in septic shock, n (% of total septic shock per MTS category)***^a^	*0 (0)*	*4 (66.7)*	*9 (69.2)*	*10 (71.4)*	*9 (69.2)*	*32 (68.1)*	*p=1.98*
***Sepsis + septic shock diagnosis/total ICU admissions, %***	*0/13 (0)*	*11/63 (17.5)*	*18/260 (6.9)*	*19/54 (35.2)*	*25/167 (15.0)*	*73/557 (13.1)*	*p=4.53*
***Mortality of sepsis patients, n (% of total sepsis per MTS category)***	*2 (22.2)*	*5 (11.1)*	*5 (20.0)*	*4 (8.9)*	*5 (27.8)*	*21 (14.8)*	*p=0.29*
***Mortality of septic shock patients, n (% of total septic shock per MTS category)***	*0 (0)*	*0 (0)*	*4 (30.8)*	*5 (35.7)*	*1 (7.7)*	*10 (21.3)*	*p=1.08*
***Positive blood culture in sepsis, n (% of total sepsis per MTS category)***	*8 (88.9)*	*32 (71.1)*	*20 (80.0)*	*25 (55.6)*	*15 (83.3)*	*100 (70.4)*	*p=0.09*
***Positive blood culture in septic shock patients, n (% of total septic shock per MTS category)***	*1 (100)*	*3 (50)*	*6 (46.2)*	*9 (64.3)*	*5 (38.5)*	*24 (51.1)*	*p=0.52*

^a^ICU admission is defined as ICU admission within 24 h after triage in ED

### Digital sepsis alerts

**Fig. 5:**
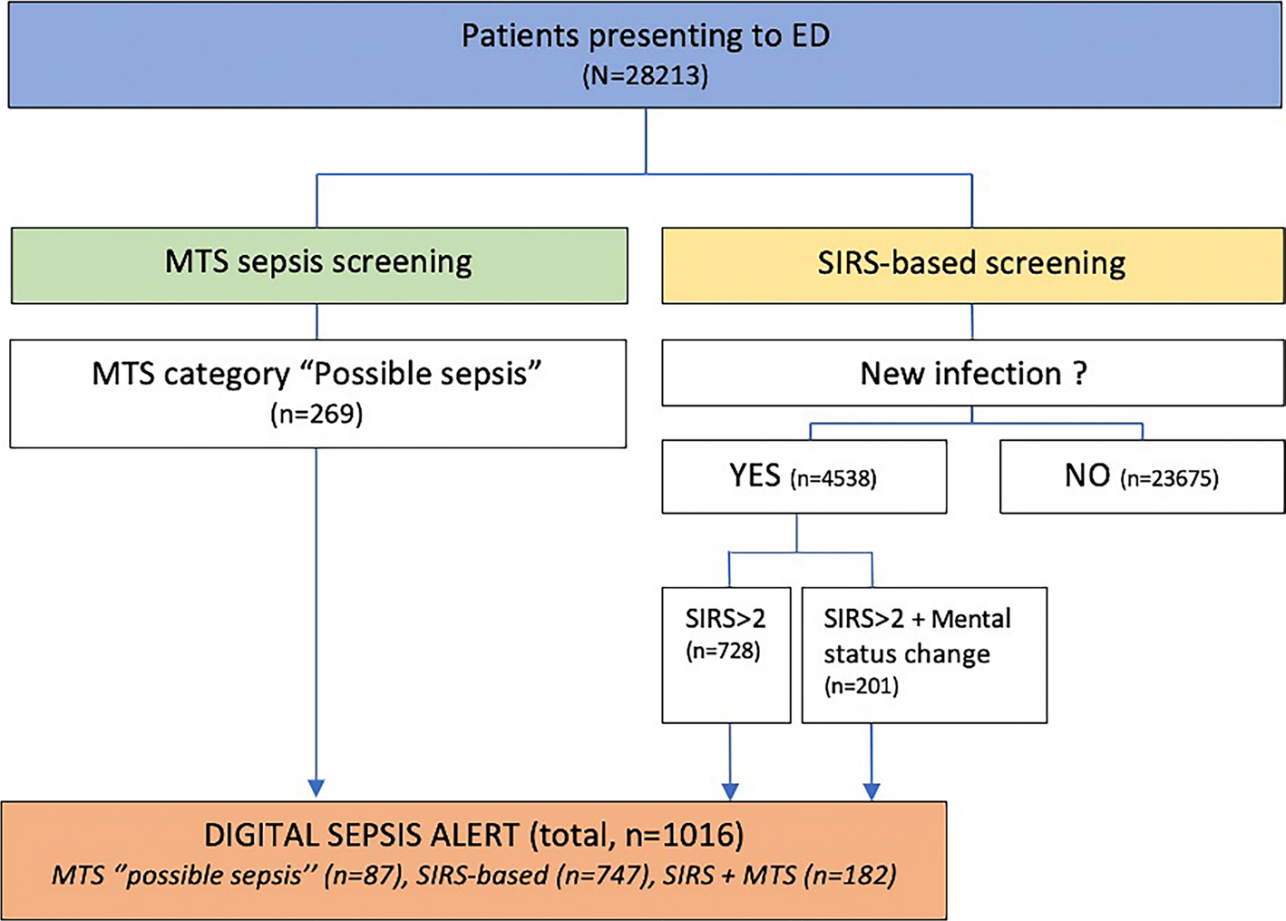
Flow chart of sepsis screening and digital sepsis alert at our center

A total of 1016 sepsis alerts were generated. SIRS-based screening accounted for most alerts and in 182 cases an alert was generated through both the MTS and SIRS-based screening. See Fig. 5.

### Performance of the Manchester triage system to detect sepsis

With the MTS to allocate sepsis patients to the specific MTS category “possible sepsis” or red category, the sensitivity was low at 47.6% (95% CI 40.3 to 55.0%). However, specificity was high at 99.4% (95% CI 99.3 to 99.5%). An absolute number of 99 patients with sepsis were not classified as “possible sepsis” and thus at risk for delay in treatment, given a negative likelihood ratio of 0.5 (95% CI 0.4 to 0.6). False-positive alerts were generated in 179 on a total of 269 alerts (66.5%) at a positive predictive value (PPV) of 33.5% (95% CI 29.0 to 38.3)

### Performance of the SIRS-based system to detect sepsis

The SIRS-based sepsis screening system showed a sensitivity of 51.9 % (95% CI 44.5 to 59.2%) and a specificity of 97.0% (95% CI 96.9 to 97.2%). A high number of false-positive alerts were generated: 831 on a total of 929 alerts at a PPV value of 10.5% (95% CI 9.2 to 12.1%).

### Performance of a combination of the MTS and SIRS-based sepsis screening

Combining the MTS and the SIRS-based screening tool showed an improved sensitivity of 64.0% (95% CI 56.7 to 70.9%) and a specificity of 96.8% (95% CI 96.6 to 97.0%). See Tables 2 and 3 for details.

**Table 2:** Performance measures of MTS, SIRS-based, and combined screening system

Test characteristic	MTS category possible sepsis	95% CI	SIRS-based system		MTS plus SIRS-based system	95% CI
**Sensitivity**	47.6%	40.3 to 55.0%	51.9%	44.5% to 59.2%	64.0%	56.7 to 70.9%
**Specificity**	99.4%	99.3 to 99.5%	97.0%	96.8% to 97.2%	96.8%	96.6 to 97.0%
**AUC**	0.74	0.73 to 0.74	0.74	0.74 to 0.75	0.80	0.80 to 0.81
**Positive likelihood ratio**	74.6	60.5 to 92.0	17.6	15.0 to 20.4	20.1	17.7 to 22.7
**Negative likelihood ratio**	0.53	0.4 to 0.6	0.50	0.43 to 0.58	0.37	0.31 to 0.46
**Positive predictive value**	33.5%	29.0 to 38.3%	10.5%	9.2 to 12.1	11.9%	10.7 to 13.3%
**Negative predictive value**	99.7%	99.6 to 99.7%	99.7%	99.6 to 99.7%	99.8%	99.7 to 99.8%
**Accuracy**	99.0%	99.0 to 99.1%	96.7%	96.5 to 96.9%	96.6%	96.4 to 96.8%

**Table 3:** Absolute numbers of triage categorization

	Sepsis	No sepsis	Total, ***n***
**MTS category “possible sepsis”**			
MTS possible sepsis	90	179	269
MTS no possible sepsis	99	27845	27944
Total, *n*	189	28024	28213
**SIRS-based screening**			
SIRS positive	98	831	929
SIRS negative	91	27193	27284
Total, *n*	189	28024	28213
**MTS plus SIRS-based system**			
Digital sepsis alert	121	895	1016
No digital sepsis alert	68	27129	27197
Total, *n*	189	28024	28213

An absolute number of 68 patients with sepsis did not give an alert in our digital patient overview with a negative likelihood ratio of 0.37 (95% CI 0.31 to 0.45).

This tool’s false-positive alerts were 895 on a total of 1016 alerts (88.1%), given a PPV of 11.9% (95% CI 10.7 to 13.3%).

### Comparison of test performance measures

The sensitivity of the combination of the screening tools was significantly higher than the MTS screening system alone. Although specificity was only slightly lower, this was also significant (McNemar’s test for sensitivity and specificity *p*<0.0001).

### Audit of triage categorization to MTS category “possible sepsis”

We chose a pragmatic, orientating approach for the audit in which the SIRS-based screening tool was used as a benchmark, knowing our SIRS-based tool inherently carries the risk of misinterpretation. We believe, however, this approach can reveal important discrepancies and flaws in the input of important data.

The presence of a new infection was scored in 4538 patients in the SIRS-based screening tool. See also Fig. 5. Of these patients, mental status change was scored in 251 patients (see checkbox Fig. 4). Blood pressure recordings and respiratory rate were cross-checked with data in the EHR.

**Fig. 6:**
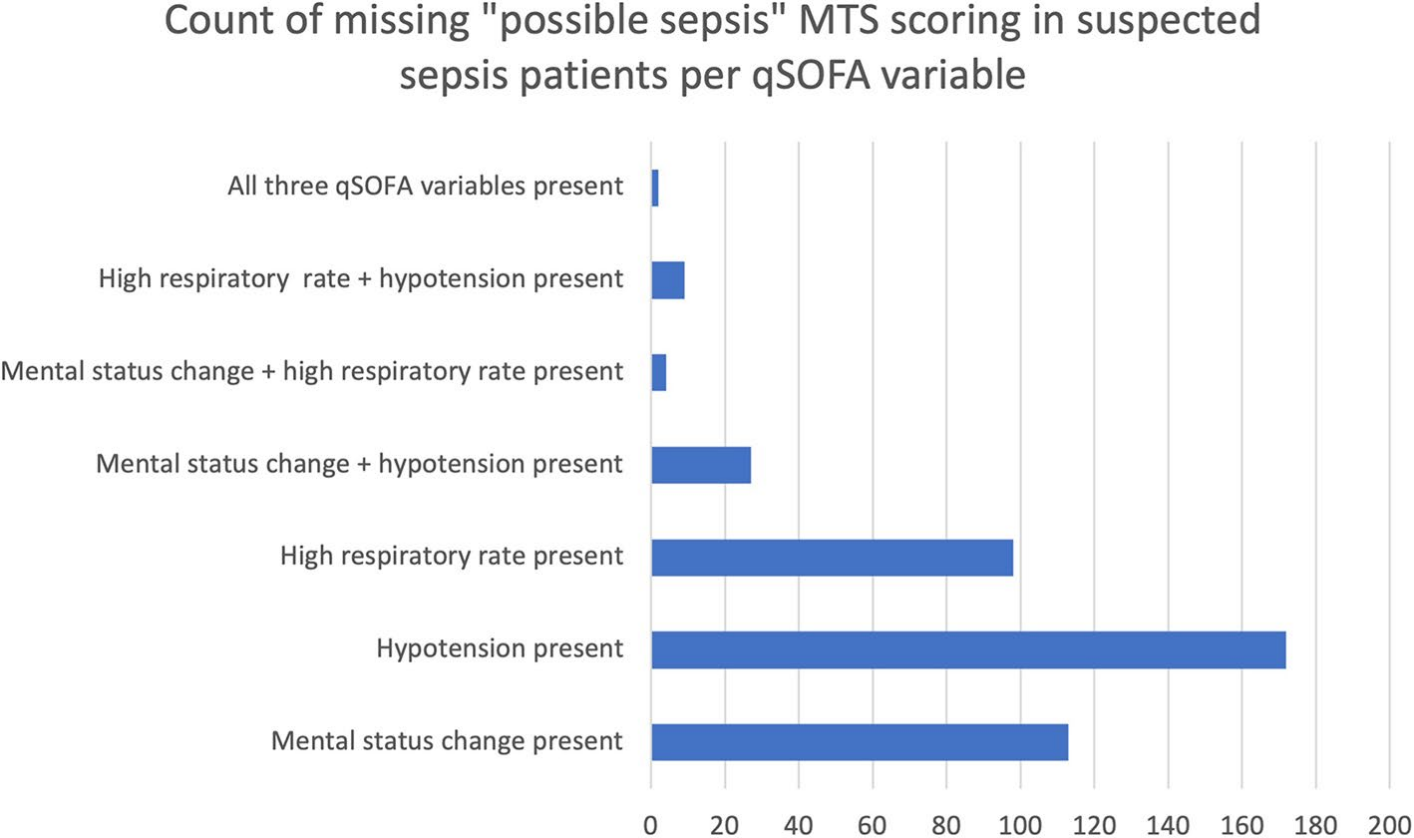
Missing MTS “possible sepsis” scoring

In this group of patients with a suspicion of infection (*n*=4538), a total of 425 patients were not assigned to “possible sepsis” in the MTS screening tool, although one or more qSOFA criteria were present. See Fig. 6.

On the other hand, on the total of 269 cases categorized to the “possible sepsis” category using the digital MTS tool, no single qSOFA criterium was present in 172 (63.9%) cases, which we could define as “overtriage” according to the definition of “possible sepsis” in the MTS. This means only 97 (36.1%) cases of the patients assigned to the possible sepsis category were correctly triaged using our audit approach.

### Characteristics of missed diagnosis of sepsis by Manchester triage

Although our study was not designed nor powered to analyze the outcome of missed cases of sepsis, we explored the individual health records of these patients.

Four out of the 17 patients with a missed diagnosis by the MTS that died during hospital stay suffered incorrect scoring of qSOFA criteria. Of the missed cases of sepsis, not a single patient did not receive antibiotics in the ED. However, no valid data on the exact timing of the administration of antibiotics was available.

## Discussion

The possible sepsis discriminator in the current version of the MTS inherently uses the qSOFA criteria. However, comparable to our results, two published studies found qSOFA score had a low sensitivity (50.2% and 47.2%, respectively) and relatively high specificity (78.1% and 69.5%, respectively) for diagnosing sepsis-3 in the emergency department [20, 21].

A combination of the MTS and a SIRS-based sepsis screening tool, a method already suggested in previous research, showed an overall fair performance in our retrospective data set [22].

The number of false-positive alerts of our digital sepsis alert (895 false-positive alerts on a total of 1016 alerts) seems acceptable in practical terms. However, harm from false-positive alerts may include missing alternative diagnoses due to early anchoring on sepsis and the subsequent effects of early, aggressive fluid intervention [23]. More research is needed to confirm these assumptions.

We were surprised by the high rate of poor application of the triage system in our data set. Human error leads to over- and underestimating scores of clinical prediction tools, which makes validating these triage systems difficult. One study investigated the effect of redesigning an electronic triage interface to make data entry less effortful [24]. Documentation of correct respiratory rate more than doubled following the interface change in this study. Importantly, we recommend taking measures to monitor the correct application of this triage tool and to adapt the user interface of the digital screening tools to minimize human error. In our case, the input of vital sign data during triage (respiratory rate, mental status change, and blood pressure) should trigger the MTS “possible sepsis” discriminator.

Mirhagi et al. showed in their meta-analysis that agreement is higher for the latest version of MTS among nurse experts [25]. However, most studies do not mention the version of the MTS reviewed and in some cases the MTS is locally adapted, rendering comparison difficult.

To overcome this problem, software companies should acquire MTS Accreditation. To get accredited, a product should achieve the MTS IT specification and update its software within the timeframes outlined by MTS.

Interestingly, machine learning algorithms are being developed that could accurately predict sepsis ahead of time [26–28]. A recent prospective, multi-site study shows a reduced mortality rate, organ failure, and length of stay after implementation of a machine learning-based early warning system, indicating early warning systems have the potential to improve sepsis patient outcomes [29].

### Strengths

One of the strengths of this study includes the rigorous iteration to screen our dataset for a definitive diagnosis of sepsis to avoid selection bias. For example, if we had only reviewed patients based on suspicion of infection in the ED as described by Seymour et al. [13], patients who were undertriaged and patients without a sepsis workup in the ED admitted to ICU within 48 h because of sepsis would not have been included.

### Limitations

Limitations of this study include the retrospective design of the study. In addition, our dataset suffered from missing data with a possible significant influence on the results. However, we took a maximum effort to verify important data points on an individual patient level.

Our hospital uses NEWS as a standard early warning system, so it would be interesting to include this system in a comparative analysis.

No definite conclusions can be made regarding the clinical implications of the performance of these sepsis screening tools.

The COVID-19 pandemic started during data collection, and our study was not designed to analyze the impact of this disease on the performance of our triage tool. However, a preliminary analysis showed that many COVID-19 patients triaged as having possible sepsis ended up with a diagnosis of COVID-19 pneumonia without sepsis.

## Conclusion

The combination of the recently updated version of the Manchester Triage System (MTS) with a specific discriminator for possible sepsis and a SIRS-based screening tool appears to have an overall acceptable performance in the early detection of sepsis.

Important discrepancies between input of data in the locally developed digital screening tools were present. Hospitals using the MTS should accredit their software solutions to avoid flaws in software design.

Future research should facilitate the implementation of machine learning techniques to detect sepsis and other life-threatening pathology and investigate the clinical implications of screening systems in the emergency department.

## Data Availability

The datasets used and/or analyzed during the current study are available from the corresponding author on reasonable request.

## Abbreviations

ED: Emergency department
SIRS: Systemic inflammatory response syndrome
(q)SOFA: (quick) Sequential Organ Failure Assessment
MTS: Manchester triage system
EHR: Electronic health record
